# Review of Mental Capacity Assessments and Best Interest Documentation on the East Suffolk Older People’s Ward at the Norfolk and Suffolk Foundation Trust

**DOI:** 10.1192/bjo.2026.11811

**Published:** 2026-06-30

**Authors:** Oluwaseun Olaluwoye, Shamim Ruhi

**Affiliations:** 1 Norfolk and Suffolk NHS foundation Trust, Norwich, United Kingdom; 2 Norfolk and Suffolk NHS foundation Trust, Ipswich, United Kingdom

## Abstract

**Aims:**

The Mental Capacity Act (MCA) 2005 requires healthcare professionals to assess a patient’s capacity to make decisions about their treatment and care. When a patient lacks capacity, a best interest assessment (BIA) must be completed to guide safe and lawful decision-making. Ensuring timely and accurate completion of these forms is essential for safeguarding patient rights and maintaining compliance with legal and Trust-wide standards.

The aim of the audit is to evaluate the completeness and timeliness of Mental Capacity Assessments (MCA) and Best Interest Assessments (BIA) completed for all new admissions to older people’s Ward between 1 January and 30 June 2025, in alignment with the MCA 2005 and local Trust policy.

**Methods:**

A retrospective review of the Trust’s patient information system was conducted to identify all admissions to older people’s Ward during the study period. Transfers from other mental health wards were excluded, and readmissions were counted once. For each admission, the following were recorded: admission status (informal, MHA sections, or DoLS), whether an MCA was completed within 24 hours, capacity outcome, and whether a required BIA was completed within 24 hours. Copies of completed MCA and BIA forms were reviewed for completeness and quality of documented rationale. Data were recorded and analysed using Microsoft Excel.

**Results:**

Thirtytwo patients were admitted during the study period. Most admissions were under section 2 of the Mental Health Act (75%), with additional admissions under section 3 (6%), DoLS (3%), and informal status (16%). MCA forms were completed within 24 hours for 84% of admissions, while 16% were not completed in the required timeframe. One patient (3%) required a BIA; however, this was not completed within 24 hours. All completed MCA forms demonstrated full completion of required sections with clear rationales documented.

**Conclusion:**

The majority of patients admitted to older people’s Ward received timely and welldocumented capacity assessments; however, delays remain in a minority of cases, including the timely completion of BIAs. These findings highlight the need for improved consistency in meeting the 24hour documentation requirement and suggest potential areas for staff training, workflow improvement, and strengthened oversight to ensure full compliance with the Mental Capacity Act 2005.

